# *In vitro* anticoagulant and antioxidant activities of *Jatropha gossypiifolia* L. (Euphorbiaceae) leaves aiming therapeutical applications

**DOI:** 10.1186/1472-6882-14-405

**Published:** 2014-10-20

**Authors:** Juliana Félix-Silva, Thiago Souza, Rafael Barros Gomes Camara, Bárbara Cabral, Arnóbio Antônio Silva-Júnior, Ivanise Marina Moretti Rebecchi, Silvana Maria Zucolotto, Hugo Alexandre Oliveira Rocha, Matheus de Freitas Fernandes-Pedrosa

**Affiliations:** Laboratório de Tecnologia & Biotecnologia Farmacêutica (TecBioFar), Departamento de Farmácia, Centro de Ciências da Saúde, Universidade Federal do Rio Grande do Norte, Rua Gal. Gustavo Cordeiro de Farias, s/n, Petrópolis, CEP 59012-570 Natal, RN Brazil; Laboratório de Biotecnologia de Polímeros Naturais (BIOPOL), Departamento de Bioquímica, Centro de Biociências, Universidade Federal do Rio Grande do Norte, Campus Universitário, 3000, Lagoa Nova, CEP 59072-970 Natal, RN Brazil; Laboratório de Farmacognosia, Departamento de Farmácia, Centro de Ciências da Saúde, Universidade Federal do Rio Grande do Norte, Rua Gal. Gustavo Cordeiro de Farias, s/n, Petrópolis, CEP 59012-570 Natal, RN Brazil; Laboratório de Hematologia Clínica, Departamento de Análises Clínicas e Toxicológicas, Centro de Ciências da Saúde, Universidade Federal do Rio Grande do Norte, Rua Gal. Gustavo Cordeiro de Farias, s/n, Petrópolis, CEP 59012-570 Natal, RN Brazil

**Keywords:** *Jatropha gossypiifolia*, Euphorbiaceae, "*Pinhão-roxo*", "Bellyache bush", Anticoagulant, Antioxidant, Cytotoxicity, Fibrinolytic, Thin layer chromatography

## Abstract

**Background:**

*Jatropha gossypiifolia* L. (Euphorbiaceae) is a medicinal plant largely used in folk medicine. Teas from the leaves are popularly used as an antithrombotic agent and the branches are frequently employed as a "thick blood" agent. Considering that the anticoagulant activity associated with antioxidant properties could be beneficial for various cardiovascular diseases, this study’s aim is the evaluation of anticoagulant and antioxidant activities of *J. gossypiifolia* leaves, seeking new therapeutic purposes for this plant.

**Methods:**

The aqueous leaf crude extract (CE) was prepared by decoction and was fractionated by liquid-liquid partition with solvents of increasing polarity. The phytochemical analysis was performed by thin layer chromatography (TLC) and by the spectrophotometric quantification of sugars, proteins and phenolic compounds. The anticoagulant activity was evaluated by prothrombin time (PT) and activated partial thromboplastin time (aPTT) tests. The capacity to act in the fibrinolytic system (fibrinolytic and fibrinogenolytic activities) was also assessed. The antioxidant activity was evaluated by total antioxidant capacity, reducing power, copper chelating activity, iron chelating activity, hydroxyl radical scavenging activity and superoxide radical scavenging assays. The potential toxicity was evaluated using hemolytic assay and the 3-(4,5-dimethylthiazol-2-yl)-2,5-diphenyltetrazoliumbromide (MTT) assay on HEK-293 cells.

**Results:**

CE showed significant anticoagulant activity in aPTT test, while no action was observed in PT test, suggesting a preferential action toward the intrinsic and/or common pathway of coagulation. No effect was observed in the fibrinolytic system. Using the aPTT test, it was observed that the residual aqueous (RA) fraction was the most active, being two times more active than CE. RA presented very significant antioxidant activity in all models tested comparable to or even higher than CE. Regarding the safety, CE and RA did not produce significant cytotoxicity in both tests employed. Phytochemical analysis revealed the presence of alkaloids, flavonoids, proteins, tannins, steroids and/or terpenoids and sugars.

**Conclusions:**

CE and RA possessed significant anticoagulant and antioxidant activity and absence of cytotoxic effect *in vitro*, thus showing the potential of the plant, especially RA fraction, as a new source of bioactive molecules for therapeutic purposes, with particular emphasis on the treatment of cardiovascular diseases.

## Background

The use of plants with medicinal purposes for the prevention and/or treatment of diseases is one of the most ancient forms of primary health care [1, 2]. Plants produce several secondary metabolites that present many important biological activities. Anticoagulant and antioxidant activities could be highlighted amongst these.

Anticoagulant drugs are needed for the short-term treatment of arterial and venous thrombotic disorders and for the long-term prevention of recurrences [3]. Although heparin has been the mainstay of anticoagulant treatment for acute thrombotic disorders for decades, this drug presents some limitations related to its clinical application, such as inefficacy in antithrombin deficient patients, bleeding complications, potential for the development of heparin-induced thrombocytopenia, immunosuppression and osteoporotic effect with long-term application as side effects [3, 4]. So, the search for new substances with anticoagulant and antithrombotic activities is relevant [4]. Medicinal plants have historically been the first source of anticoagulant and antithrombotic molecules [5].

Reactive oxygen species (ROS) are free radicals naturally produced in the cells and are involved in many cellular biochemical activities such as signal transduction, gene transcription and regulation of soluble guanylate cyclase activity [6, 7]. However, overproduction of free radicals or failure in endogenous antioxidant mechanisms can cause oxidative damage to biomolecules (lipids, proteins and DNA), eventually leading to many chronic diseases, such as atherosclerosis, cardiovascular diseases, cancer, diabetes, rheumatoid arthritis, post-ischemic perfusion injury, myocardial infarction, chronic inflammation, stroke, septic shock, aging and other degenerative diseases in humans [6, 7]. Antioxidants are important substances that have the ability to protect the organism from the damage caused by the oxidative stress. Due to this ability, there is a special interest in the presence of natural antioxidants in medicinal plants that may help the organism to maintain the normal balance of ROS [7, 8]. Plants are frequently reported as a good source of antioxidant components, such as phenolic compounds [9].

Atherosclerosis is a vascular disease characterized by the accumulation of fatty substances, cholesterol, cellular waste products, calcium and other substances in the arterial wall, and involves an inflammatory response to the local low-density lipoprotein (LDL) [9, 10]. The oxidation of LDL is a well-described phenomenon in atherosclerosis, and ROS are involved in the pathophysiology of this disease [6]. Among the injuries that may occur during atherosclerosis progression, the most severe consequence is the promotion of thrombous formation, generated by the activation of platelet aggregation and blood coagulation [11]. Thus, compounds with anticoagulant and antioxidant can be used in the actual medicine for treatment of atherosclerosis.

*Jatropha gossypiifolia* L. is a medicinal plant belonging to the Euphorbiaceae family, popularly known in Brazil as "pinhão-roxo" or known worldwide as "bellyache-bush". This species is widely distributed in countries of tropical, subtropical and dry tropical weather and tropical semi-arid regions of Africa and Americas [12]. Several human and veterinary uses in traditional medicine are described for different parts (leaves, stems, roots, seeds and latex). Preparations based on this plant, such as infusion, decoction and maceration, by oral or topical routes, are frequently used. The most frequent reports relate to its antihypertensive, anti-inflammatory, antiophidic, analgesic, antipyretic, antimicrobial, healing, haemostatic, anti-anemic and antidiabetic applications, among other examples [13–15]. An important feature of *J. gossypiifolia* species is that, due to its important potential medicinal applications, it is included in the National List of Medicinal Plants of Interest to Brazilian Public Health System (*Relação Nacional de Plantas Medicinais de Interesse ao Sistema Único de Saúde Brasileiro* – *RENISUS*), which is a report published by the Brazilian Health Ministry that includes 71 species of medicinal plants that have the potential to generate pharmaceutical products of interest to public health [16].

Regarding the chemical compounds ascribed to this species, alkaloids, coumarins, flavonoids, lignoids, phenols, saponnins, steroids, tannins and terpenoids were already detected in different extracts from different parts of this plant [17]. Among the main activities already studied for this species (including many kinds of extracts from different parts of the plant), the antihypertensive, antineoplasic, antimicrobial, antiophidic and anti-inflammatory activities mainly stand out, supporting some of its popular uses [15, 18, 19].

The present study was carried out aiming to evaluate the anticoagulant and antioxidant activities of the aqueous leaf extract of *J. gossypiifolia.* Using a bioguided fractionation, the main fraction responsible for the anticoagulant activity was identified (residual aqueous fraction). This fraction also possessed significant antioxidant activity *in vitro* and absence of cytotoxic effect *in vitro*, thus showing the potential of the plant and, more specifically, of this fraction as a new source of bioactive molecules for therapeutic purposes, with particular emphasis on the treatment of cardiovascular diseases such as atherosclerosis, since anticoagulants and antioxidants are used in the treatment of these disorders.

## Methods

### Chemicals and reagents

Luteolin, orientin, isoorientin, vitexin, isovitexin, D-glicose, gallic acid, Cremophor® EL (polyoxyl 35 castor oil), 3-(4,5-dimethylthiazol-2-yl)-2,5-diphenyltetrazoliumbromide (MTT), *Bothrops jararaca* snake venom, bovine fibrinogen, bovine serum albumin, ascorbic acid, pyrocatechol violet and ferrozine were purchased from Sigma-Aldrich (St. Louis, MO, USA). All reagents of sodium dodecilsuphate polyacrylamide gel electrophoresis were purchased from GE Healthcare (Piscataway, NJ, USA). All reagents used for cell culture procedures were purchased from Cultilab (Campinas, SP, Brazil). All other reagents and solvents used were of analytical grade. The water used was purified by reverse osmosis.

### Preparation of pool of plasma and red blood cell (RBC) suspension

After written informed consent has been obtained, blood from adult healthy volunteers free from medication for at least two weeks and fasted for at least 8 h was taken by venipuncture and collected into 0.105 M sodium citrate (9:1 v/v, blood: anticoagulant) and K_3_EDTA (1,5 mg EDTA: 1 mL blood) tubes (BD Vacutainer®, Franklin Lakes, NJ, USA). The human plasma pool was prepared from the supernatants obtained after centrifugation at 800 g for 10 min at room temperature of the citrated blood. The plasma was used up to two weeks after being obtained. For red blood cell (RBC) suspension preparation, blood collected with EDTA was centrifuged at 560 g for 10 min at room temperature and the red blood cell pellet was subsequently rinsed three times with PBS. A 20% (v/v) RBC suspension was obtained by dilution with PBS. The RBC was used immediately after preparation. The procedures for human blood collection were approved by the Ethics Committee in Human Research from Federal University of Rio Grande do Norte (protocol no. 092/09).

### Plant material

Leaves of *Jatropha gossypiifolia* L. (Euphorbiaceae) were collected in Carnaubais, municipality of Rio Grande do Norte State, Brazil, at coordinates 36.80°W 5.27°S, in April 2012. The botanical identification of the material was performed by Msc. Alan de Araújo Roque and a voucher specimen was deposited at the Herbarium from the Centro de Biociências of Universidade Federal do Rio Grande do Norte, Brazil (UFRN 12561). The collection of the plant material was conducted under authorization of Brazilian Authorization and Biodiversity Information System (SISBIO) (process number 35017) and Brazilian Access Authorization and Dispatch Component of Genetic Patrimony (CGEN) (Process 010844/2013-9). The leaves were dried at room temperature for about 18 days, triturated with an industrial blender and stored in hermetically sealed bottles away from light and humidity until use for extract preparation.

### Extract preparation

Dried leaves were submitted to decoction (1:10 w/v, plant: solvent) for 15 min at a temperature of around 100°C to obtain the aqueous leaf extract of *J. gossypiifolia,* named crude extract (CE). CE, obtained after vacuum filtration, was freeze-dried and one part of it was dissolved in PBS at adequate concentrations for the biological assays.

### Crude extract (CE) fractionation

CE was fractionated by liquid-liquid partition with solvents of crescent polarity in order to obtain the dichloromethane (CH_2_Cl_2_), ethyl acetate (AcOEt), *n*-buthanol (BuOH) and residual aqueous (RA) fractions. The fractions were dried and one part of them was dissolved in PBS or in Cremophor® 5% solution in PBS (according to fraction solubility), at adequate concentrations for the biological assays.

### Phytochemical analysis

For phytochemical characterization of CE and fractions, they were submitted to thin layer chromatography (TLC) analysis and had the content of sugar, protein and phenolic compounds determined spectrophotometrically.

#### Thin Layer Chromatography (TLC)

CE and fractions were analyzed by TLC using aluminium pre-coated sheets with silica gel F_254_ (Merck®, Darmstadt, Germany) as adsorbent. Three mobile phases were employed: (1) ethyl acetate: formic acid: water (8:1:1 v/v/v), (2) toluene: ethyl acetate: formic acid (5:5:0.5 v/v/v) and (3) *n*-butanol: acetic acid: water (3:1:1 v/v/v). The chromatograms were analyzed under 254 and 365 nm UV light and different spray reagents were used according to the metabolite investigated (sulfuric vanillin + heating, natural product reagent + UV 365 nm, ferric chloride, Dragendorff reagent and ninhindrin + heating). The retention factors (*Rf*), color and behavior of the spots were recorded for further comparison with chromatographic profiles of reference substances from the literature in the area [20]. Standard samples of flavonoids were employed for co-TLC analysis.

#### Total content of sugars, proteins and phenolic compounds

Total sugar was estimated by Dubois method using D-glicose as standard [21]. Phenolic compounds were determined by Folin-Ciocalteu’s method using gallic acid as standard [22]. The Bradford method was used for protein quantification using bovine serum albumin (BSA) as standard [23].

### Coagulation and fibrinolysis assays

The action of CE and fractions on haemostatic system was assessed by the evaluation of its anticoagulant activity in prothrombin time (PT) and activated partial thromboplastin time (aPTT) tests, as well as by its capacity to act in fibrinolytic system.

#### Prothrombin Time (PT) test

The action in extrinsic pathway was evaluated by PT test, as previously described in literature, with a few modifications [24]. The test was carried out using commercial reagent kits (CLOT Bios Diagnostica, São Paulo, SP, Brazil). Plasma (90 μL) was mixed with 10 μL of samples (0.1 – 2 μg/μL) and incubated at 37°C for 5 min at 37°C. Then, 200 μL of PT assay reagent (rabbit brain extract and calcium chloride) pre-warmed at 37°C for 10 min was added and the clotting time was recorded by a digital coagulometer ("Laser Sensor" Clotimer, CLOT, São Paulo, SP, Brazil). Plasma alone (only with vehicle) was used as control (absence of anticoagulant activity). Heparin (1 IU/mL) (Cristalia®, Itapira, SP, Brazil) was used as positive control.

#### Activated partial thromboplastin time (aPPT) test

The action in intrinsic and common pathways was evaluated by aPTT test, as previously described in literature, with a few modifications [24]. The test was carried out using commercial reagent kits (CLOT Bios Diagnostica, São Paulo, SP, Brazil). Plasma (90 μL) was mixed with 10 μL of samples (0.1 – 2 μg/μL) and incubated at 37°C for 5 min at 37°C, before the addition of pre-warmed aPTT reagent (rabbit brain extract and ellagic acid) and incubation at 37°C for 2 min. Pre-warmed (37°C) 25 mM calcium chloride was then added and the clotting time recorded by a digital coagulometer ("Laser Sensor" Clotimer, CLOT, São Paulo, SP, Brazil). Plasma alone (only with vehicle) was used as control (absence of anticoagulant activity). Heparin (1 IU/mL) (Cristalia®, Itapira, SP, Brazil) was used as positive control.

#### Fibrinolytic activity

The fibrinolytic activity was determined as previously described in literature, with a few modifications [25, 26]. First, 100 μL of citrated human plasma was mixed with equal volume of 100 mM CaCl_2_ and incubated at 37°C for 120 min for fibrin clot formation. The plasma fibrin clot was washed 5 times with PBS and then incubated with different concentrations of samples (0.1 – 2 μg/μL) at 37°C for 120 min. 100 μL of 0.0625 M Tris-HCl pH 6.8 containing 10% v/v glycerol, 10% v/v β-mercaptoethanol, 2% w/v SDS and 0.05% w/v bromophenol blue was added, followed by boiling for 5 min. The samples were centrifuged at 800 g for 10 min and the supernatant was then analyzed by 7.5% SDS-PAGE [27]. Plasma fibrin clot alone was used as control, for visualization of the intact fibrin profile. *B. jararaca* venom was used as positive control for fibrinolytic activity [28].

#### Fibrinogenolytic activity

The fibrinogenolytic activity was determined as previously described in literature, with a few modifications [29]. Different concentrations of samples (0.1 – 2 μg/μL) were mixed with 50 μg of fibrinogen and then incubated for 180 min at 37°C. 1.5 M Tris-HCl pH 8.8 containing 10% v/v glycerol, 10% v/v β-mercaptoethanol, 2% w/v SDS and 0.05% w/v bromophenol blue was added, followed by boiling for 5 min. The samples were then analyzed by 12% SDS-PAGE [27]. Fibrinogen alone was used as control, for visualization of the intact fibrinogen profile. *B. jararaca* venom was used as positive control for fibrinogenolytic activity [28].

### Antioxidant activity

The antioxidant activity was evaluated by six *in vitro* models: total antioxidant capacity, reducing power, copper chelating activity, iron chelating activity, hydroxyl radical scavenging activity and superoxide radical scavenging activity.

#### Total antioxidant capacity

The assay for total antioxidant capacity was performed using the phosphomolybdenum complex formation method [30]. Tubes containing 100 μg of samples and the reagent solution (0.6 M sulfuric acid, 28 mM sodium phosphate and 4 mM ammonium molybdate) were incubated at 95°C for 90 min. Then, the mixture was cooled at room temperature and the absorbance measured at 695 nm against a blank. The total antioxidant activity was interpolated using a standard curve with known concentrations of ascorbic acid and expressed as milligram of ascorbic acid equivalent per gram of extract (AAE).

#### Reducing power

The reducing power was quantified as described previously, with a few modifications [31]. Samples (0.1 – 2 μg/μL) were added to the reaction mixture (0.2 M phosphate buffer pH 6.6 containing 1% w/v ferricyanide potassium) and incubated at 50°C for 20 min. The reaction was stopped by adding 10% w/v trichloroacetic acid solution. Then, the solution was mixed with water and 0.1% w/v ferric chloride. The absorbances were read at 700 nm. The result was expressed as the percentage of the activity shown by 0.2 μg/μL of ascorbic acid.

#### Copper chelating activity

The ability to chelate copper ions was assessed using the pyrocatechol violet method [32]. 30 μL of samples (0.1 – 2 μg/μL) were mixed with 200 μL of 50 mM acetate buffer pH 6.0, 6 μL of 4 mM pyrocatechol violet and 100 μL of 50 μg/μL copper II sulfate pentahydrate. The absorbances were read at 632 nm in a microplate reader (Epoch-Biotek®, Winooski, VT, USA). The chelating activity was expressed as the chelation percentage in relation to a blank (absence of sample). The IC_50_ (concentration that produces 50% of copper ion chelation) was calculated. EDTA was used as positive control.

#### Iron chelating activity

The ability to chelate iron ions was assessed as previously described in literature, with a few modifications [31]. Samples (0.1 – 2 μg/μL) were added to the reaction mixture containing 2 mM ferrous chloride and 5 mM ferrozine. The mixture was stirred and incubated for 10 min at room temperature. The absorbance was measured at 562 nm. The chelating activity was expressed as the chelation percentage in relation to a blank (absence of sample). The IC_50_ (concentration that produces 50% of iron ion chelation) was calculated. EDTA was used as positive control.

#### Hydroxyl radical scavenging activity assay

The scavenging activity against hydroxyl radical was measured based on Fenton’s reaction, as previously described in the literature, with a few modifications [33]. Hydroxyl radicals were generated using 150 mM sodium phosphate buffer pH 7.4 containing 10 mM ferrous sulphate, 10 mM EDTA, 2 mM sodium salicylate and 30% hydrogen peroxide with different concentrations of sample (0.1 – 2 μg/μL). In control tubes, sodium phosphate buffer replaced hydrogen peroxide. The solutions were incubated at 37°C for 60 min, and the presence of hydroxyl radicals was detected by monitoring the absorbance at 510 nm. The scavenging activity was expressed as inhibition percentage (scavenging percentage). The IC_50_ (concentration that produces 50% of hydroxyl radical scavenging) was calculated. Gallic acid was used as positive control.

#### Superoxide radical scavenging activity assay

The assay was based on the capacity of the samples to inhibit the photochemical reduction of nitroblue tetrazolium (NBT) in the riboflavin-light-NBT system, as previously described, with a few modifications [34]. The reaction mixture (50 mM phosphate butter pH 7.8, 13 mM methionine, 2 mM riboflavin, 100 mM EDTA and 75 mM NBT) and samples at different concentrations were mixed. After 10 min under illumination with a fluorescent lamp, the production of blue formazan was monitored at 560 nm. Tubes containing only the reaction mixture were maintained in the dark and served as control to reaction. The scavenging activity was expressed as inhibition percentage (scavenging percentage). The IC_50_ (concentration that produce 50% of superoxide radical scavenging) was calculated. Gallic acid was used as positive control.

### Cytotoxic activity

As a preliminary study to evaluate the potential toxicity of the plant, *in vitro* cytotoxicity studies were performed in red blood cell suspension (hemolytic activity) and in human embryonic kidney 293 (HEK-293) cells.

#### Hemolytic assay

The hemolytic assay was performed as previously described in literature, with a few modifications [35]. Briefly, 5 μL of 20% (v/v) red blood cell (RBC) suspension were incubated at 37°C for 60 min with 500 μL of samples at different concentrations (0.1 – 2 μg/μL). The mixtures were then centrifuged at room temperature for 2 min at 8,600 g and the absorbance of the supernatant was measured at 540 nm with a microplate spectrophotometer (Epoch-Biotek®, Winooski, VT, USA). Water was used as positive control (100% RBC lysis) and PBS as negative control (absence of RBC lysis). The values of treated cells were calculated as a percentage of the positive control.

#### In vitro *cytotoxicity against human embryonic kidney cells (HEK-293)*

Human embryonic kidney 293 cells (HEK-293) (ATCC® CRL-1573) were cultured under standard conditions in DMEM (Dulbecco’s modified Eagle’s medium) supplemented with fetal bovine serum (FBS) at a final concentration of 10%. Cells were maintained in cell culture flasks at 37°C in a humidified atmosphere containing 5% CO_2_ and were collected by treatment with trypsin. Cells (1 × 10^4^ cells per well) were seeded in medium supplemented with FBS (10%) and cultured for 24 h in 96-well microplates to promote adhesion. The following day, the medium was removed and replaced with fresh medium free of FBS. Serum deprivation was used for synchronizing cell cycle. The next day, the medium was replaced with fresh medium with FBS (10%) containing serial dilutions of the samples (3.9 – 1000 μg/mL) previously sterilized in a 0.45 μm membrane. The negative control was exposed to the standard medium supplemented with FBS (10%) without sample. After 24 h, the MTT assay was performed as a marker of cell viability, as previously described in literature [36]. Briefly, medium containing extract was replaced with medium containing 1 μg/μL of MTT and incubated for 4 h at 37°C. After incubation, the supernatant was removed and the purple formazan crystal formed was solubilized in ethanol, stirred for 15 min and the absorbance was measured at 570 nm in a microplate reader (Epoch-Biotek®, Winooski, VT, USA). The absorbance of the negative control (no sample) was considered as 100% cell viability and the values of treated cells were calculated as a percentage of the negative control.

### Statistical analysis

The results were expressed as mean ± SEM with n = 3. One-way ANOVA with Tukey’s post test or Student’s t-test, as well regression analysis were performed using GraphPad Prism version 5.00 for Windows, GraphPad Software, San Diego California USA. p values less than 0.05 were considered significant.

## Results and discussion

Antithrombotic drugs are pivotal in the prevention and/or treatment of thrombotic disorders. Secondary metabolites from vegetal origin are a potential source of anticoagulant drugs [5]. Its popular use as well as some interesting studies in literature prompted us to evaluate the anticoagulant activity of *J. gossypiifolia*.

Teas obtained by decoction of the leaves of *J. gossypiifolia* are popularly used as an antithrombotic agent [37] and the branches are frequently employed as a "thick blood" agent, a popular claim related to antiplatelet action [38]. Although there is no study in literature about the possible anticoagulant activity of this plant, a previous study showed the potentiality of the juice extracted from the fresh leaves of *J. gossypiifolia* as an anticoagulant for haematological analyses, with an efficiency comparable to conventional laboratory anticoagulants [39]. Therefore, with the aim of therapeutic purposes, the *in vitro* anticoagulant action of the aqueous leaf extract of *J. gossypiifolia* was investigated in the present study.

**Figure 1:**
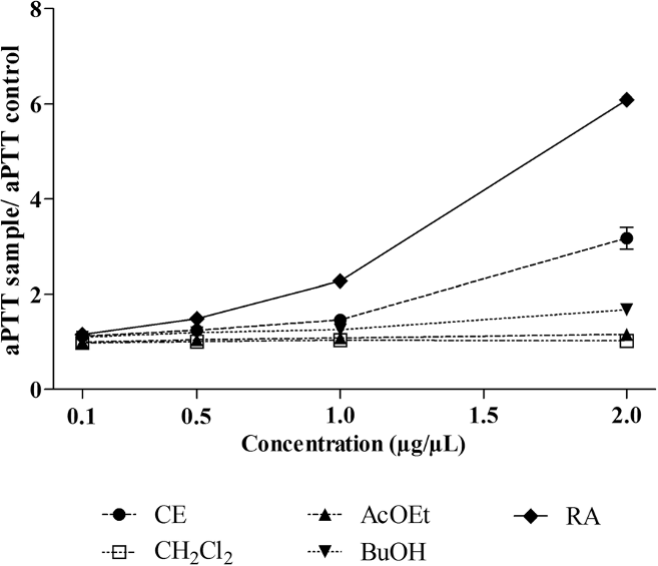
**Anticoagulant activity of crude extract (CE) and fractions from** ***Jatropha gossypiifolia*** **on aPTT test.** CE: crude extract. CH_2_Cl_2_: dichloromethane fraction. AcOEt: ethyl acetate fraction. BuOH: *n*-butanol fraction. RA: residual aqueous fraction. Values expressed as mean ± SEM with n = 3. CE, AcOEt, BuOH and RA, in all concentrations tested, presented p < 0.05 when compared to control (absence of sample) by Tukey’s test (ANOVA).

The anticoagulant activity of the crude extract of *J. gossypiifolia* (CE) was evaluated by the prothrombin time (PT) and activated partial thromboplastin time (aPTT) assays, using normal citrated human plasma. As can be observed in Figure 1, CE was able to prolong the clotting time in aPTT test by up to 3 times, demonstrating its anticoagulant activity. In PT test, no prolongation of the clotting time was observed (results not shown). The prolongation of aPTT indicates the inhibition of the intrinsic and/or common pathway of coagulation, whereas no prolongation of PT demonstrates no inhibition of the extrinsic pathway [24]. So, the present results suggest that CE inhibits preferentially intrinsic and/or common pathways of coagulation. Heparin was used as positive control and as expected presented significant anticoagulant activity, with PT higher than 60 s (negative control: 16.27 ± 0.32 s) and aPTT higher than 240 s (negative control: 35.07 ± 0.03 s).

In order to identify the constituents of CE responsible for the anticoagulant activity presented, a phytochemical screening by thin layer chromatography (TLC) was performed. But first, the CE was fractionated by liquid-liquid partition to obtain fractions with different polarities and thus facilitate the chromatographic analysis of the compounds. The yields are presented in Table 1. As can be observed in the Table 1, the fractions with higher yield are the more polar ones (BuOH and RA fractions), thus suggesting that in CE there is a predomination of polar over apolar compounds.

**Table 1:** **Yields of extraction and fractionation process of** ***Jatropha gossypiifolia*** **leaves**

Crude extract/ fractions	Yield (%), in relation to	
	dried leaves*	crude extract**
CE	13.57	100
CH_2_Cl_2_ fraction	0.53	3.87
AcOEt fraction	0.43	3.19
BuOH fraction	3.67	27.07
RA fraction	3.92	28.85

CE: crude extract. CH_2_Cl_2_: dichloromethane fraction. AcOEt: ethyl acetate fraction. BuOH: *n*-butanol fraction. RA: residual aqueous fraction. *yield calculated based on initial amount of dried leaves used in the preparation of the crude extract (50 g). **yield calculated based on amount of equivalent dried CE use for fractionation (6.79 g).

The chromatographic analysis of CE and fractions of *J. gossypiifolia* with specific spray reagents indicated the presence of alkaloids, terpenes and/or steroids, phenolic compounds, flavonoids, tannins and amines.

According to TLCs performed and by comparison with the literature, CH_2_Cl_2_ fraction presented green-fluorescent bands when revealed with natural product reagent under UV 365 nm, suggesting the presence of flavonoids, as well brownish bands with Dragendorff’s reagent, suggesting the presence of alkaloids.

In the AcOEt fraction, treatment with ferric chloride revealed several dark bands suggestive of phenolic compounds. The presence of tannins can also be suggested, detectable as pink bands by sulfuric vanillin under heating that revealed as black bands with ferric chloride but are not visualized with natural product reagent. By co-TLC analysis, employing as a mobile phase ethyl acetate: formic acid: water (8:1:1, v/v/v) and revelation with natural product reagent under UV 365 nm, the presence of some bands with *Rf* (retention factor) and coloration characteristics of the following flavonoids could be observed in AcOEt fraction: isoorientin (*Rf* = 0.4, dark yellow-orange band), isovitexin (*Rf* = 0.45, green fluorescent band), orientin (*Rf* = 0.52, yellow band) and vitexin (*Rf* = 0.62, green fluorescent band). A band with *Rf* and coloration characteristics of the flavonoid luteolin (*Rf* = 0.54, yellow fluorescent band) was also observed by co-TLC in this fraction, employing the mobile phase toluene: ethyl acetate: formic acid (5:5:0.5, v/v/v) and revelation with natural product reagent under UV 365 nm.

The BuOH fraction TLC analysis revealed only flavonoids, since all bands revealed with vanillin were revealed with natural product reagent. By co-TLC, some bands were observed that were similar to those detected in AcOEt fraction: isoorientin, orientin and vitexin.

In RA fraction it was not possible to visualize the presence of clear bands when the plate was developed with the spray reagents, except for the presence of a brownish zone at the point of application in the TLC plate revealed with sulfuric vanillin, which could indicate the presence of sugars; a black zone at the point of application when revealed with ferric chloride, which is indicative of phenolic compounds; and the presence of a purple zone with ninhidrin, which could indicate the presence of amino acids, peptides and/or proteins. In view of the possible presence of sugars, phenolic compounds and proteins, as well as the lack of more concrete clues about the compounds present in RA fraction, the content of these compounds was determined in this fraction and in CE, as shown in Table 2.

**Table 2:** **Content of sugars, phenolic compounds and proteins of crude extract (CE) and residual aqueous fraction (RA) from** ***Jatropha gossypiifolia***

Sample	Sugars (%)	Phenolic compounds (%)	Proteins (%)
CE	20.0 ± 0.3	18.7 ± 1.5	2.4 ± 0.4
RA fraction	15.6 ± 0.6	15.2 ± 1.0	3.7 ± 0.3

Values expressed as mean ± SEM with n = 3.

Based on these dosages, the presence of phenolic compounds and sugars could be confirmed. In addition, it could be visualized that proteins represent only a low percentage of the crude extract composition.

With the exception of luteolin, the other flavonoids have already been identified in the leaves of *J. gossypiifolia* [40, 41]. For the genus *Jatropha*, luteolin was described previously only in the species *Jatropha unicostata* [42]. Additionally, to the best of our knowledge, there are no phytochemical studies regarding the use of water as solvent for the extraction of *J. gossypiifolia* constituents. This is important to be noted since popular use occurs more frequently with infusions or decoctions, and thus, little is known about the constitution of this type of extract. Furthermore, more commonly, the studies with *J. gossypiifolia* presented in the literature use solvents or mixtures of solvents with non polar characteristics, which could contribute to further characterization mainly of non polar compounds such as terpenoids and lignoids. Polar compounds such as flavonoids, tannins and sugars are poorly described in the species so far [12, 17].

Giving continuity to the assessment of the anticoagulant activity of *J. gossypiifolia*, the four fractions obtained from CE were evaluated by the aPTT and PT test. In relation to PT, no alteration was observed with any of the fractions (results not shown). On the other hand, in the aPTT test, interesting results were obtained, as can be observed in Figure 1. Once again, an action preferentially towards the intrinsic and/or common pathway of coagulation could be suggested for aqueous leaf extract of *J. gossypiifolia*, since any of the fractions presented prolongation of PT, but some of them prolonged the aPTT. These active fractions in aPTT were the most polar ones: BuOH and RA. The AcOEt presented statistically significant prolongation of aPTT (p < 0.05), however, this anticoagulant activity was not significant from the biological point of view. The BuOH fraction presented anticoagulant activity, but this was weaker than the CE. The RA fraction was the most active, prolonging the aPTT by up to 6 times, being 2 times more active than CE. Based on this, it is possible to conclude that this fraction contains the main compounds responsible for the anticoagulant action observed in the aqueous leaf extract of *J. gossypiifolia*.

In addition to anticoagulant activity, CE and RA were also tested in relation to their capacity to hydrolyze fibrin and fibrinogen, in view of investigating its potentiality as a thrombolytic agent. However, in both tests employed, no effect was observed (results not shown).

These are interesting results since it show that the anticoagulant activity observed may be due an inhibitory action upon clotting factors, and not only to a simple degradation (proteolytic action) of the proteins involved in the coagulation cascade. Additionally, the fact that only aPTT was prolonged shows that the inhibitory effect is not due to a simple chelation of calcium ions, since if it was true, the PT should be prolonged too, what was not observed. One possible hypothesis for the anticoagulant action observed is the presence of protease inhibitors in CE, especially serine proteases inhibitors, since the coagulation is constituted, basically, of a cascade of proteolytic enzymes. In fact, Félix-Silva et al. [18] showed that the aqueous leaf extract of *J. gossypiifolia* was able to inhibit several enzymatic and biological activities induced by *Bothrops jararaca* snake venom, including the inhibition of proteolytic activity of this venom, the inhibition of procoagulant activity upon a solution of crude fibrinogen (which is attributed to serine proteases thrombin-like from snake venom) and *in vivo* inhibition of the hemorrhagic activity (which is attributed to hemorrhagic metaloproteases from snake venom), showing so that this extract has the ability to inhibit snake venom proteases.

Having in mind the potentiality of CE and, mainly the RA active fraction, as well as considering that the anticoagulant activity associated with antioxidant properties could be beneficial for various cardiovascular diseases, the antioxidant of CE and the RA active fraction were also investigated.

Phenolic compounds are commonly found in both edible and non edible plants, and they have been reported to have multiple biological effects, including antioxidant activity. Extracts rich in phenolics are increasingly of interest to the pharmaceutical and food industry [9]. As discussed before in this paper, we could observe in both CE and RA a great presence of these compounds, which was another factor that prompted us to test the antioxidant activity of these products.

The term "antioxidant" refers to compounds that can prevent the formation of biological substances and chemical oxidation damage induced by reactive species, such as ROS. The formation process of these reactive species occurs through a chain reaction involving three steps (initiation, propagation and termination) wherein the antioxidants act through several mechanisms. Thus, different methods were used to evaluate the effect of CE and RA fraction at the different stages of initiation (e.g. total antioxidant capacity and reducing power), propagation (e.g. chelation of copper and iron ions) and termination (e.g. scavenging of superoxide and hydroxyl radicals) [43].

**Figure 2:**
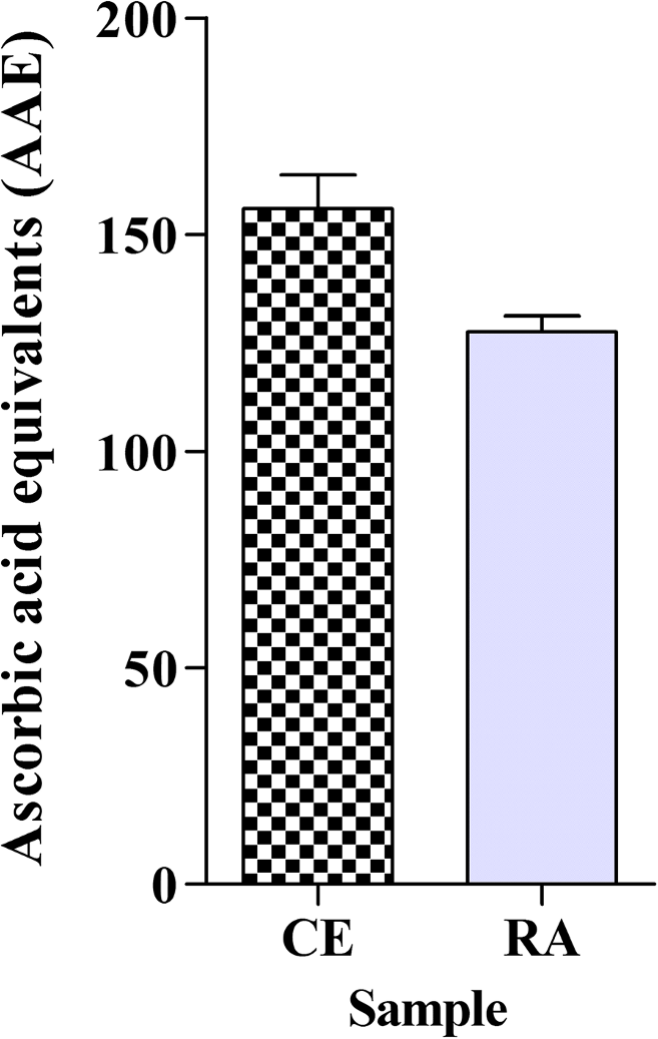
**Total antioxidant capacity of crude extract (CE) and residual aqueous fraction (RA) from** ***Jatropha gossypiifolia*** **.** The results are expressed as milligram of ascorbic acid equivalents per gram of sample (AAE). Values expressed as mean ± SEM with n = 3.

The total antioxidant capacity test evaluates the ability of a sample to donate electrons, thus neutralizing free radicals such as ROS. As can be observed in Figure 2, both CE and RA fraction presented significant antioxidant activity in this test. The RA fraction, however, presented an antioxidant activity significantly (p < 0.05) lower than CE, at about 18%. The values obtained by both CE and RA was higher than the values obtained in the same experiment with the plant *Plukenetia volubilis,* which presented values ranging from 59.31 to 97.76 mg/g of AAE, according to the extractor solvent used for extract preparation [8]. Thus, the detected values obtained in the present study were extremely interesting, which prompted us to conduct further antioxidant tests to determine the potential antioxidant mechanisms of the *J. gossypiifolia* CE and RA fraction.

**Figure 3:**
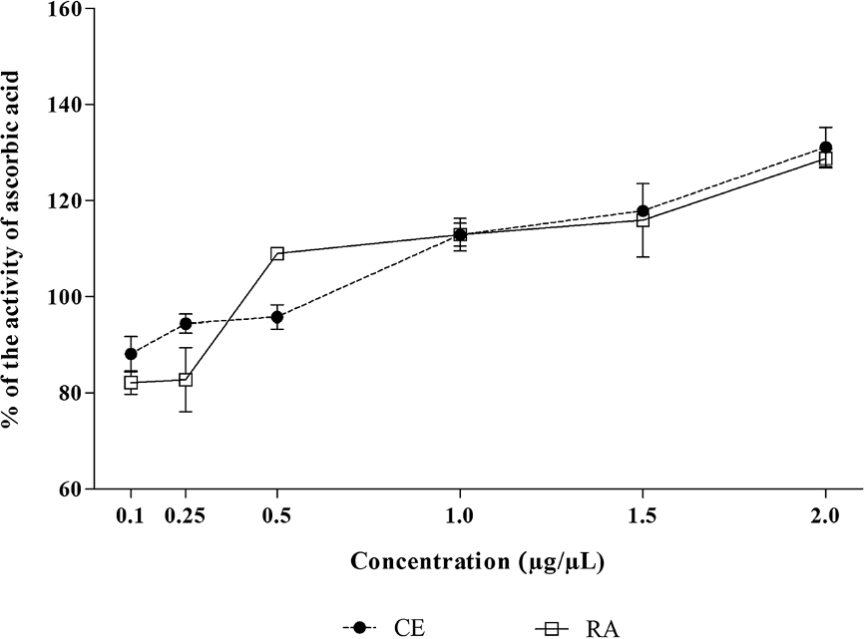
**Reducing power of crude extract (CE) and residual aqueous fraction (RA) from** ***Jatropha gossypiifolia*** **.** The results are expressed as percentage of reducing activity equivalent to ascorbic acid in a concentration of 0.2 μg/μL. Values expressed as mean ± SEM with n = 3.

The reducing power test evaluates the capacity of a sample to donate electrons. The result of this test was expressed in reducing activity equivalent to ascorbic acid at a concentration of 0.2 μg/μL. It was observed that both CE and RA presented highly significant reducing power, as can be visualized in Figure 3. The activity obtained was very similar for CE and RA (p > 0.05). As can be observed in the Figure 3, at 1 and 0.5 μg/μL, CE and RA, respectively, presented reducing power higher than the ascorbic acid at 0.2 μg/μL (reducing activity in equivalents of 0.2 μg/μL ascorbic acid higher than 100%), demonstrating a promising result for antioxidant activity. The reducing power of compounds seems to function as an inhibitor of chain reactions of free radicals by means of donation of electrons, since this activity is mediated by redox reactions [43].

**Figure 4:**
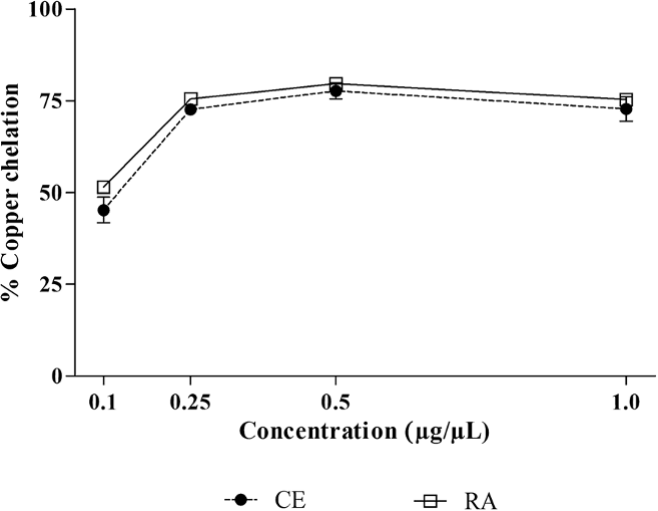
**Copper chelating activity of crude extract (CE) and residual aqueous fraction (RA) from** ***Jatropha gossypiifolia*** **.** The results are expressed as percentage of copper chelation. Values expressed as mean ± SEM with n = 3.

The equilibrium of the concentration of copper ions in biological systems is crucial for the regulation of cellular functions. When an increase in its concentration occurs, there is an increase in the production of reactive oxygen species, due in large part to Fenton and Haber-Weiss reactions. In addition, through the Fenton reaction, the preformed lipid hydroperoxides are decomposed to form alkoxyl radicals, strong oxidizing agents which can propagate the chain reaction of lipid peroxidation or react with other cellular constituents. Consequently, the chelation of copper ions may be crucial for the prevention of the production of reactive species that damage the target biomolecules [44, 45]. Therefore, the copper ions chelating effect of CE and RA fraction from *J. gossypiifolia* was evaluated. As can be observed in Figure 4, both CE and RA fraction presented significant copper chelating activity, reaching up to about 70% of copper chelation. Although the results were very similar in CE and RA fraction, statistic significance was observed for 0.1, 0.25 and 1 μg/μL concentrations (p < 0.05), being the RA fraction slightly better than CE.

**Figure 5:**
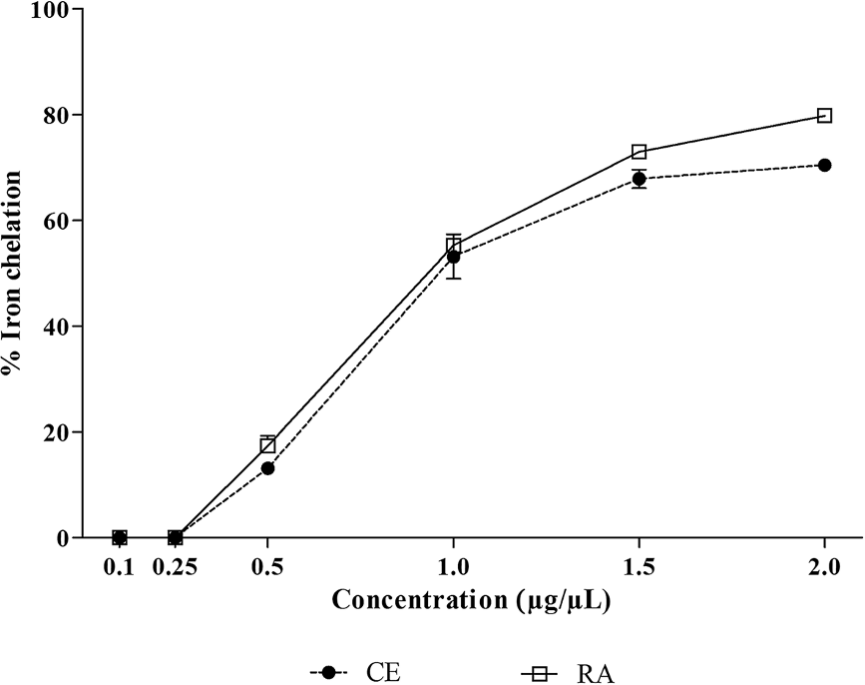
**Iron chelating activity of crude extract (CE) and residual aqueous fraction (RA) from** ***Jatropha gossypiifolia*** **.** The results are expressed as percentage of iron chelation. Values expressed as mean ± SEM with n = 3.

The iron chelating effect is very important since it inhibits the interaction between lipids and metals by forming insoluble metal complexes with ferrous ions. Furthermore, it is an effective way to eliminate the generation of hydroxyl radicals since it prevents iron from interacting with hydrogen peroxide, thus preventing the decomposition of hydrogen peroxide and the formation of an even more damaging free radical [43]. As can be observed in Figure 5, CE and RA fraction presented significant iron chelating activity, reaching up to about 80% of chelation. Although in smaller concentrations the activity shown by CE and RA fraction was very similar, at 2 μg/μL RA fraction was significantly more active, RA being about 12% more active than CE (p < 0.001).

**Figure 6:**
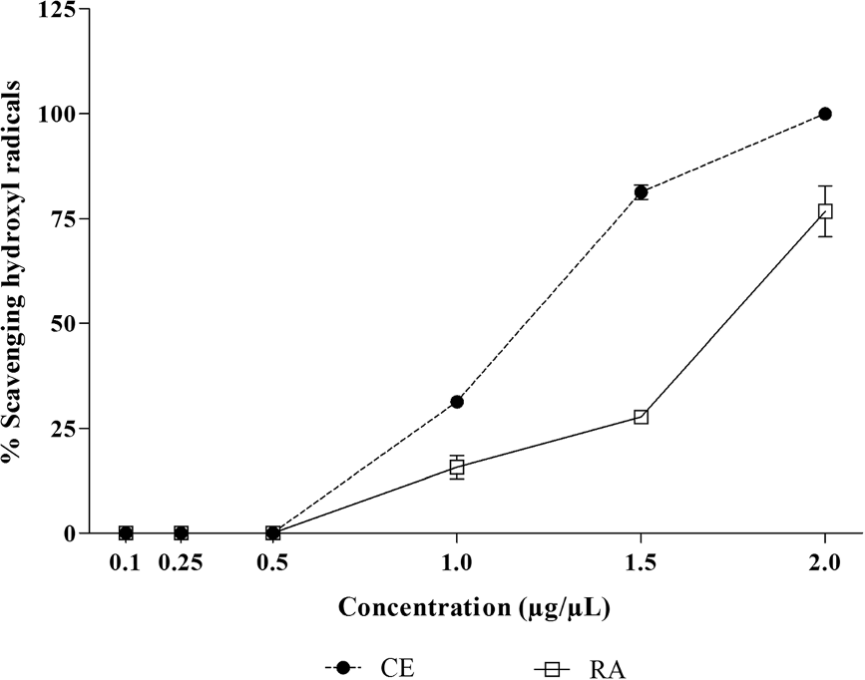
**Hydroxyl radical scavenging activity of crude extract (CE) and residual aqueous fraction (RA) from** ***Jatropha gossypiifolia*** **.** The results are expressed as percentage of scavenging of hydroxyl radicals. Values expressed as mean ± SEM with n = 3.

**Figure 7:**
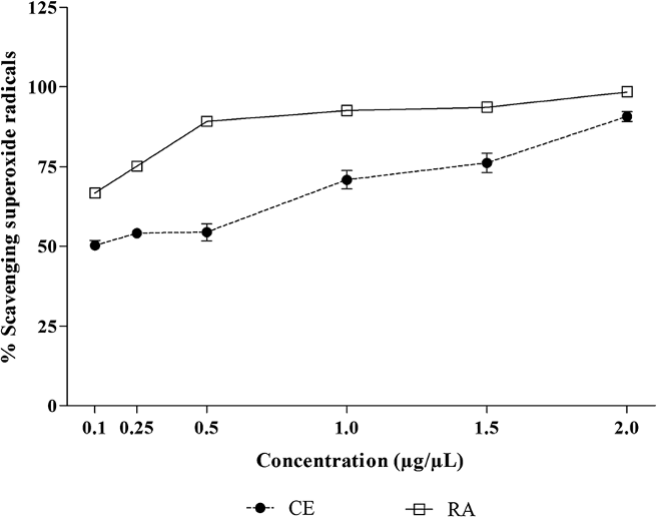
**Superoxide radicals scavenging activity of crude extract (CE) and residual aqueous fraction (RA) from** ***Jatropha gossypiifolia*** **.** The results are expressed as percentage of scavenging of superoxide radicals. Values expressed as mean ± SEM with n = 3.

Hydroxyl radicals and superoxide anions are ROS implicated in cell damage. The hydroxyl radical is the most reactive of the radicals, making it extremely harmful. Its main source of production *in vivo* is due to the reaction of transition metals with the superoxide ion by the Fenton reaction. On the other hand, superoxide anion is considered a primary ROS, capable of generating reactive derivatives by direct interaction with other molecules or by means of processes catalyzed by metals or enzymes also being produced within the mitochondria. Due to the harmful effect on the body, these ROS are associated with numerous diseases, such as strokes, cancer, diabetes, liver, and neuronal lesions [6, 7, 46]. In view of this, the radical scavenging activity of CE and RA fraction from *J. gossypiifolia* was evaluated. As can be observed in Figures 6 and 7, both CE and RA fraction presented significant radical scavenging ability. In relation to hydroxyl radicals, although no inhibition was observed in lower concentrations (up to 0.5 μg/μL), at the higher concentrations, CE and RA presented very high scavenging ability. CE was able to scavenge 100% of the hydroxyl radicals. This effect was higher than that observed in RA fraction (p < 0.001), which achieved at most about 75% of scavenging ability of hydroxyl radicals. On the other hand, in the superoxide radical scavenging activity assay, from the lowest concentrations, both CE and RA showed high scavenging ability, and RA fraction was more active than CE (p < 0.001).

Table 3 summarizes the results obtained in the antioxidant activity tests, showing the IC_50_ values for CE and RA fraction. In general, RA fraction was more active (lower IC_50_ value) than CE, with the exception of the hydroxyl radical scavenging ability, as already discussed. The lower IC_50_ for RA in superoxide radical scavenging ability (about 5 times lower than CE) strongly suggests that this fraction contains the main compounds responsible for the referred activity observed in the aqueous leaf extract of *J. gossypiifolia.*

**Table 3:** **IC**_**50**_ **of crude extract (CE) and residual aqueous fraction (RA) from** ***Jatropha gossypiifolia*** **in antioxidant assays**

Activity	IC _50_ (μg/μL)		
	CE	RA	Standard
Copper chelation	0.111	0.081	0.017 (EDTA)
Iron chelation	1.104	1.034	0.011 (EDTA)
Hydroxyl radical scavenging	1.205	1.673	0.293 (gallic acid)
Superoxide radical scavenging	0.118	0.025	0.003 (gallic acid)

IC_50_: concentration that presents 50% of the referred activity.

The antioxidant activity of extracts from *J. gossypiifolia* was also evaluated earlier by Kharat et al. [47]. In this work, firstly, the high content of phenols, tannins and flavonoids in the leaves prompted the authors to evaluate the antioxidant activity of the leaves. DPPH free radical, ferric thiocyanate and nitric oxide scavenging methods were used to analyze the *in vitro* antioxidant activity of methanol, ethyl acetate and aqueous extracts, demonstrating positive results. The authors attributed the free radical scavenging activity to the presence of flavonoids [47]. On the other hand, a study showed that different extracts (petrol ether, chloroform, ethyl acetate and *n*-butanol) from whole plant of *J. gossypiifolia* had only partial antioxidant activity in DPPH scavenging, total antioxidant capacity and lipid peroxidation tests [48]. From these extracts, the ethyl acetate was the most active, which correlated positively with its higher content of phenolic compounds in comparison with the other extracts [48].

Considering that *Jatropha* species are known to be toxic and considering previous studies that showed that ethanol extracts from *J. gossypiifolia* aerial parts exhibited noticeable toxicity [12, 49], *in vitro* cytotoxicity studies were performed as a preliminary method to evaluate the potential toxicity of CE and the active fraction RA.

The potential hemolytic activity of CE and RA was investigated by measuring the lysis of a 20% (v/v) human red blood cell (RBC) suspension in a spectrophotometric lysis assay. CE and RA were tested at different concentrations (up to 2 μg/μL) and no significant red blood cell lysis was observed (results not shown). Red blood cells hemolysis is characterized by the breakdown of the red blood cell membrane leading to the release of hemoglobin into the surrounding plasma. This deleterious event can be caused by a large number of conditions and can lead to anemia and hypoxia. The osmotic fragility of the red blood cell is classically used as an *in vitro* assay to evaluate the effects of chemicals on cell membrane [35, 50].

The *in vitro* cytotoxicity of CE and RA was evaluated in cell culture against human kidney epithelial cell line (HEK-293 cells) by MTT assay, which measures indirectly the cellular viability by quantification of the amount of blue formazan crystals, which are the product from the cleavage of MTT by cells presenting active mitochondria [36]. The results showed that at any concentration tested (up to 1 μg/μL) CE and RA were not cytotoxic, with a viability percentage similar to the control cells (treated only with medium in absence of sample) (results not shown).

The results suggest that even with the concentration of the bioactive compounds in the RA fraction, there was no increase in the toxicity of this fraction in relation to CE. It is important to note that both RBC and HEK-293 are human cells, which can reinforce the absence of cytotoxicity by CE and RA. These results suggest that the aqueous extract of the leaves, compared to the ethanol extract of the aerial parts tested by Mariz et al. [51] may be less toxic possibly due to an eventual difference in chemical composition that may have occurred, taking into account both the different plant parts and extractor solvent used for the different preparation of the extracts. In fact, a study investigating the acute oral toxicity of an aqueous leaf extract of *J. gossypiifolia* showed no sign of toxicity in rats in doses up to 2,000 mg/kg [52].

In conclusion, the presented results showed that *J. gossypiifolia* leaves, especially RA fraction, have significant beneficial effects as an anticoagulant and antioxidant agent. In addition, the absence of *in vitro* cytotoxicity against human cells could reinforce its therapeutic potential. Thus, this study shows the potential of this plant as a new source of bioactive molecules for therapeutic purposes.

## Conclusions

The results shown in this work demonstrate that the aqueous leaf extract of the vegetal species *Jatropha gossypiifolia* has significant anticoagulant activity. Using a bioguided fractionation assay, it was possible to conclude that the fraction responsible for the activity in the crude extract was the residual aqueous fraction. In addition to the anticoagulant activity, this fraction also proved to be a good source of antioxidant compounds. Since compounds with anticoagulant and antioxidant can be used in current medicine for treatment of various cardiovascular diseases, we suggest that based in our results, the aqueous leaf extract of *J. gossypiifolia*, specially its residual aqueous fraction, shows promising potential as a future therapeutic agent. In addition, the absence of *in vitro* cytotoxicity against human cells could reinforce its potential. Thus, the results presented here could give scientific evidence, at least partially, for the popular use of this plant in complementary and alternative medicine and show its potential as a new source of bioactive molecules for therapeutic purposes.

## Authors’ information

MFFP and HAOR are CNPq fellowship-honored researchers.

## Abbreviations

AcOEt: Ethyl acetate fraction of *J. gossypiifolia* crude extract
aPTT: Activated partial thromboplastin time
BuOH: *n*-butanol fraction of *J. gossypiifolia* crude extract
CE: *J. gossypiifolia* crude extract
CH_2_Cl_2_: Dichloromethane fraction of *J. gossypiifolia* crude extract
EDTA: Ethylenediamine tetraacetic acid
HEK-293: Human embryonic kidney 293 cells
LDL: Low-density lipoprotein
MTT: 3-(4,5-dimethylthiazol-2-yl)-2,5-diphenyltetrazoliumbromide
PBS: Phosphate buffered saline
PT: Prothrombin time
RA: Residual aqueous fraction of *J. gossypiifolica* crude extract
RBC: Red blood cell suspension
ROS: Reactive oxygen species
TLC: Thin layer chromatography.
